# Cardiological Findings in Children and Adolescents Before and After Guanfacine Treatment for Attention Deficit and Hyperactivity Disorder

**DOI:** 10.3390/children12030302

**Published:** 2025-02-27

**Authors:** Bekir Yükcü, Bedia Sultan Önal, Cansu Çobanoğlu Osmanlı, Gülsüm Yitik Tonkaz, Berkan Şahin

**Affiliations:** 1 Department of Pediatric Cardiology, Giresun Maternity and Children Training and Research Hospital, Giresun 28200, Türkiye; 2 Department of Child and Adolescent Psychiatry, Giresun University Faculty of Medicine, Giresun 28200, Türkiye; bedia.onal@giresun.edu.tr (B.S.Ö.); cansu.cobanoglu@giresun.edu.tr (C.Ç.O.); gulsum.yitiktonkaz@saglik.gov.tr (G.Y.T.); berkan.sahin@giresun.edu.tr (B.Ş.)

**Keywords:** ADHD, guanfacine, cardiovascular safety, electrocardiography

## Abstract

**Objective:** This study evaluates the short-term cardiovascular effects of guanfacine treatment in children and adolescents with attention deficit/hyperactivity disorder (ADHD). The treatment’s impact on novel electrocardiographic parameters was also investigated. **Methods:** In a retrospective study conducted between January 2023 and June 2024, 37 patients aged 6–18 years with ADHD underwent baseline and follow-up cardiac evaluations including electrocardiography (ECG) and blood pressure measurements. Novel ECG markers (QRS-T angle, QT dispersion, QTc dispersion, Tp-e interval, Tp-e dispersion, Tp-e/QT ratio, and Tp-e/QTc ratio) were analyzed alongside standard parameters such as heart rate, QT, and corrected QT (QTc) intervals. Guanfacine was initiated at 1 mg and titrated weekly until an optimal clinical response was achieved. **Results:** Guanfacine treatment led to a significant reduction in heart rate (−12.3 bpm; *p* < 0.001) and P wave axis (−12.3°; *p* = 0.038) and an increase in QT interval (+16.8 ms; *p* = 0.014). However, no significant changes were observed in blood pressure, QTc duration, or the novel ECG parameters. Importantly, the absence of any changes in these advanced markers supports the cardiovascular safety of guanfacine. Two patients experienced side effects (bradycardia and hypotension), leading to treatment discontinuation. ANCOVA analysis indicated that the duration between ECG evaluations significantly influenced the QT interval changes, emphasizing the importance of timing when monitoring cardiovascular effects. **Conclusions:** Guanfacine demonstrated minor, statistically significant effects on the selected cardiac parameters without clinically meaningful changes to or adverse impacts on the novel ECG markers investigated. As extended-release guanfacine has only been available in Türkiye for the management of ADHD for approximately two years, studies evaluating its clinical efficacy and side effects are critical for clinicians working in this field.

## 1. Introduction

Attention deficit/hyperactivity disorder (ADHD) is a neurodevelopmental disorder characterized by difficulties in maintaining attention and/or excessive hyperactivity and impulsivity that are disproportionate to the individual’s developmental level [1]. ADHD is one of the most common psychiatric disorders in childhood. Epidemiological studies report a prevalence ranging from 3.4% to 7.2%, although the rates vary across different geographic regions [2,3]. If left untreated, ADHD can lead to severe academic, social, and psychological problems throughout life. While the hyperactive symptoms tend to diminish with age, attention deficits persist into adulthood in approximately 30% to 70% of patients [4,5,6,7].

Psychostimulants such as methylphenidate (MPH) and amphetamines have been the primary treatment choice for ADHD since their introduction in 1937 [8,9]. These medications provide robust symptom control in the short term, with an efficacy rate of 65–75% [10,11]. However, 20–35% of patients may exhibit an inadequate response to psychostimulants [12]. In some cases, pre-existing psychiatric or cardiac conditions may worsen, making continued treatment challenging [13,14,15]. For these reasons, researchers have turned to alpha-2 agonists (α2As) in resistant cases where patients show partial or no response to stimulants, aiming to achieve higher remission rates [16,17,18,19,20,21,22,23]. Additionally, α2As have emerged as alternative primary treatments due to their lower incidence of side effects such as weight loss, nausea, vomiting, and hepatotoxicity [24,25]. Guanfacine exerts its effects by activating α2A receptors in the prefrontal cortex, reducing presynaptic calcium channel activity; this, in turn, decreases norepinephrine levels, thereby modulating guanfacine’s therapeutic efficacy [26,27,28]. Controlled studies with extended-release guanfacine (GXR) have demonstrated response rates of 50–65% during the acute treatment phase [16,20]. Numerous clinical studies have demonstrated that guanfacine is effective in the treatment of ADHD and generally well tolerated: most side effects are mild and/or transient and do not require discontinuation of the medication [17,20,29,30,31]. The most commonly reported side effects include somnolence, sedation, fatigue, upper abdominal pain, dry mouth, nausea, dizziness, weight gain, constipation, and postural dizziness [32].

Guanfacine exerts its cardiovascular effects by antagonizing sympathetic activity, resulting in outcomes such as hypotension and bradycardia. Unlike stimulants, guanfacine is associated with slight reductions in heart rate (~8 beats per minute [bpm]) and systolic blood pressure [BP] (~10 mmHg). These effects are generally considered clinically insignificant and are reported to return to baseline values upon the discontinuation of treatment [16]. Studies on guanfacine have reported no electrocardiographic (ECG) abnormalities as serious adverse effects during either monotherapy or combination therapy [20,33]. Guanfacine is an effective option for adjunctive therapy or monotherapy alongside stimulant medications. Due to its lack of significant cardiac effects, it is supported for safe use in children and adolescents. Although numerous studies have consistently indicated the absence of severe cardiac problems associated with guanfacine, the available data remain limited due to confounding factors and the complexities of combination therapies.

Previous studies evaluating the effects of guanfacine have predominantly focused on parameters such as heart rate, QT interval, QTc duration, and BP [16,34]. However, emerging novel ECG markers such as the T-wave and QRS axis, QRS-T angle, QT-QTc and Tp-e dispersion, Tp-e interval, Tp-e/QT ratio, and Tp-e/QTc ratio have gained attention for their value in assessing ventricular repolarization and electrical instability [35,36]. These markers provide valuable insights into both the global and transmural dispersion of repolarization, which is critical for predicting the risk of severe ventricular arrhythmias and sudden cardiac death (SCD). The Tp-e interval indicates the total dispersion of ventricular repolarization and was closely linked to arrhythmogenesis [35,36]. Additionally, the Tp-e/QT ratio is increasingly recognized as a reliable indicator of repolarization abnormalities. Moreover, it is less affected by variables such as heart rate and body weight [36].

This study aims to evaluate the short-term cardiological outcomes of treating children and adolescents with ADHD using guanfacine, a medication that has been actively used in Türkiye over the past year. Additionally, the authors hope that this study contributes to a better understanding of guanfacine’s cardiovascular safety profile by utilizing novel ECG parameters not included in previous research.

## 2. Materials and Methods

### 2.1. Study Population

This retrospective study was conducted between January 2023 and June 2024 at Giresun Maternity and Children’s Health Training and Research Hospital, Türkiye. The study included patients aged 6–18 years who were referred to the Pediatric Cardiology Clinic for cardiovascular evaluation before initiating guanfacine treatment, as those with any positive findings in the pre-treatment cardiac risk assessment were directed for further evaluation. All patients were previously diagnosed with ADHD by psychiatrists trained to assess children and adolescents. Guanfacine treatment was initiated at a starting dose of 1 mg in all patients and titrated weekly until an optimal clinical response was achieved. The initial cardiac evaluation (heart rate, BP, ECG) was performed before starting the 1 mg dose, and the final cardiac evaluation was conducted two weeks after reaching the optimal dose. Patients initiated on guanfacine treatment were those who had an insufficient response to stimulant or atomoxetine treatment or were unable to tolerate these treatments at optimal doses due to side effects. In our study, the doses of psychotropic medications previously used by patients who were initiated on guanfacine as adjunct therapy remained unchanged. Since these medications were maintained at stable doses, they were assumed to have no significant impact on cardiac parameters. Therefore, any observed clinical and electrocardiographic changes in these patients were attributed solely to the cardiac effects of guanfacine.

Demographic information, including age, gender, weight, height, and body mass index (BMI), along with clinical data such as current medications, the presence of comorbid conditions, additional medications used, systolic and diastolic BP values, ECG, and echocardiography results, were obtained from patient files. BMI was categorized according to the definitions provided by the World Health Organization (WHO) [37].

### 2.2. Inclusion and Exclusion Criteria

#### 2.2.1. Inclusion Criteria

- Patients aged between 6 and 18 years.
- Diagnosed with ADHD according to the DSM-IV diagnostic criteria.
- The diagnosis was established using the semi-structured diagnostic interview Kiddie Schedule for Affective Disorders and Schizophrenia—Present and Lifetime Version (K-SADS-PL) and a clinical interview conducted by a specialist in child and adolescent psychiatry.
- A Clinical Global Impression—Severity (CGI-S) score ≥ 4 for ADHD at the time of evaluation, indicating moderate or greater symptom severity.
- Referred for a cardiac risk assessment, which included known pre-existing cardiovascular disease, symptoms suggestive of cardiovascular disease (e.g., chest pain, palpitations, syncope), and a family history of sudden cardiac death or cardiovascular disease under the age of 40.

#### 2.2.2. Exclusion Criteria

- Diagnosed with comorbid bipolar affective disorder or psychotic disorder and neurologic disorder.
- BP values outside the normal range for age and height [38].
- History of unexplained syncope episodes.
- Serious heart rhythm abnormalities (diagnosed with long QT syndrome, sinus bradycardia with a resting heart rate of <60 bpm, clinically significant bradycardia).
- Presence of medical conditions contraindicating the use of stimulants or alpha-agonist medications.
- Any other condition deemed unsuitable for the study as per clinical judgment.

**Figure 1:**
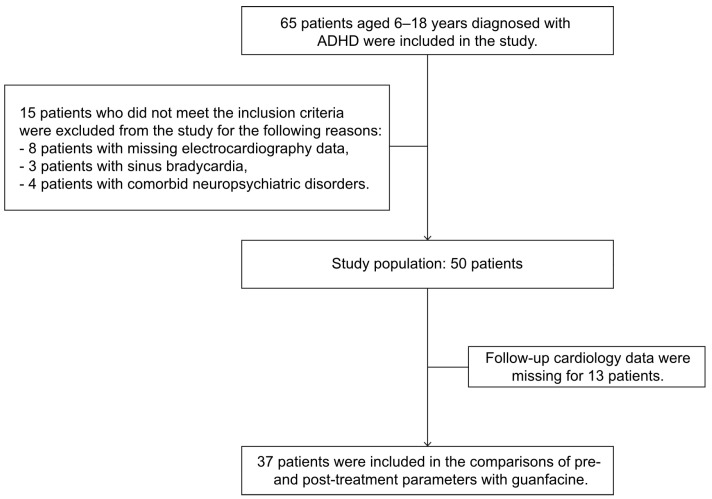
Flowchart of patient enrollment and exclusion criteria.

A total of 65 patients who met the inclusion criteria were initially enrolled in the study. Among these, eight, three, and four patients were excluded due to missing cardiology data, sinus bradycardia (heart rate < 60 bpm), and comorbid neuropsychiatric disorders, including autism spectrum disorder and bipolar disorder, respectively. Of the remaining 50 patients with complete baseline ECG and hemodynamic data, 13 were further excluded due to having missing follow-up ECG and cardiology parameters. Consequently, 37 patients were included in the final evaluation of the effects of guanfacine treatment on cardiology parameters (Figure 1).

### 2.3. Heart Rate and BP

To ensure consistency and accuracy, all measurements were performed using a validated automated device, Nihon Kohden (GE Medical Systems, Milwaukee, WI, USA). Measurements were performed while the subjects were in a stable physical and emotional state and free from acute infections, fever, pain, fear, anxiety, stress, or any other secondary factors that could influence cardiovascular readings. Care was taken to ensure that the patients had not recently engaged in physical activity, consumed caffeine, or used vasoactive medications that might affect the results. The measurements were conducted in a quiet environment, with the patients lying down and resting for at least two minutes prior to the procedure.

### 2.4. ECG Analysis

ECGs were recorded in a quiet environment with the patients lying in a supine position. Standard 12-lead ECGs were obtained using a Biocare Digital Electrocardiograph (Shenzhen Biocare Bio-Medical Equipment Co., Ltd., Shenzhen, China) device at a paper speed of 25 mm/s and a calibration of 10 mm/mV. The recordings were subsequently uploaded to the hospital’s Picture Archiving and Communication System for detailed analysis on a computer. All the measurements were performed using Portable Document Format files, which allowed for up to 15× magnification via the zoom function to ensure precise and accurate interval identification. Each interval was manually measured for three consecutive beats, and the average value was then calculated.

The following procedures were applied:

- QT interval measurement: The QT interval was measured as the time from the onset of the Q wave to the end of the T wave in the lead where the T wave morphology was most distinct.
- QTc interval measurement: QTc intervals were calculated using Bazett’s formula as follows:

QTc = QT/√RR

where QT is the measured QT interval (ms) and RR is the RR interval (ms) derived from the ECG. RR intervals were determined as the time between two consecutive R-wave peaks.

- Tp-e interval measurement: The Tp-e interval was measured as the time from the peak of the T wave to the end of the T wave. The measurements were performed in the lead where the T wave morphology was most distinct.
- Dispersions (QT, QTc, Tp-e, P): Dispersion values were calculated as the difference between the maximum and minimum interval values across all 12 ECG leads.
- PR interval: The PR intervals were manually measured as the time from the beginning of the P wave to the start of the QRS complex.
- Axes measurements (P axis, T axis, QRS axis): The P axis, T axis, and QRS axis values were automatically obtained from the ECG machine’s output and directly recorded in the study database.
- Ratios: The ratios were computed using a calculator and subsequently recorded in the data collection form.

### 2.5. Statistical Analyses

The statistical analyses were performed using IBM SPSS Statistics for Windows, version 25.0 (IBM Corp., Armonk, NY, USA). Descriptive statistics were used to present the demographic and clinical characteristics of the study population. Data normality was assessed using the Kolmogorov–Smirnov test, and all continuous variables were expressed as mean ± standard deviation. Paired samples *t*-tests were conducted to compare the pre-treatment and post-treatment variables. For the repeated measures analysis of covariance (ANCOVA), QT interval, heart rate, and P axis were selected as the dependent variables. The independent variables included guanfacine dose groups and the duration between the first and second ECG measurements (used as a covariate). Interaction terms (e.g., guanfacine dose × duration) were also included in the models to evaluate their combined effects. Box’s Test of Equality of Covariance Matrices was used to assess model assumptions, and partial eta squared values were calculated to determine the effect sizes. Statistical significance was set at *p* < 0.05. Results with a *p*-value less than 0.05 were considered statistically significant and are reported in bold in the results section.

## 3. Results

The initial evaluation included 37 patients diagnosed with ADHD, comprising 9 (24.3%) females and 28 (75.7%) males, with a mean age of 11.65 ± 2.65 years (range 6–16). The median weight was 40 kg (range 24–95), and the median height was 147 cm (range 123–173). The median BMI value was 17.3 (range 12.76–35.32). Echocardiographic results revealed normal or insignificant lesions, valvular lesions, and ascending aortic dilatation in 23 (62.2%), 13 (35.1%), and 1 (2.7%) patient, respectively. The mean interval between the initial and follow-up evaluations was 27.8 ± 16.8 days for the 37 patients who returned for the second evaluation (Table 1).

Significant reductions were observed in the heart rate and P wave axis, along with a prolongation of the QT interval. No significant changes were noted in the other parameters, including BP, PR interval, and QTc duration (Table 2).

In one patient, a 40 ms prolongation of the QTc interval and a 25 bpm reduction in heart rate (baseline heart rate: 50–54 bpm) were observed, leading to the discontinuation of guanfacine treatment. In another patient, dizziness, visual dimming, and hypotension (75/46 mmHg) were reported, resulting in the termination of guanfacine therapy (Table 3).

The post-treatment heart rate decreased by an average of 12.3 bpm compared to pre-treatment levels (*p* < 0.001). The P wave axis showed an average reduction of 12.3°, while the QT interval increased by an average of 16.8 ms post-treatment compared to baseline (Table 4).

**Figure 2:**
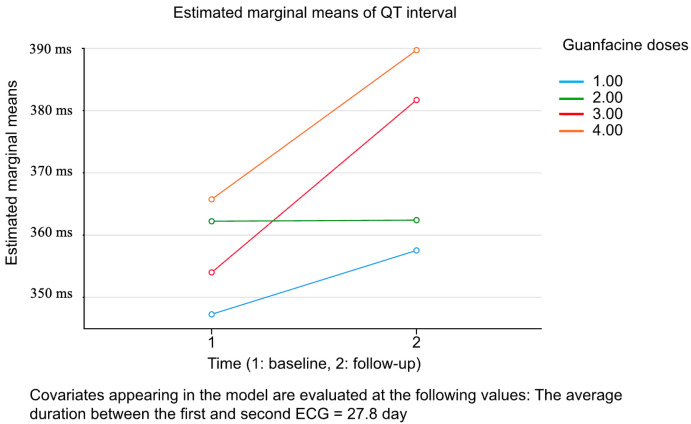
The estimated marginal means of the QT interval across baseline (1) and follow-up (2). The analysis evaluates the impact of different guanfacine dose groups (1.00 mg, 2.00 mg, 3.00 mg, and 4.00 mg) on the QT interval over time. The covariate, defined as the average duration between the first and second ECG measurements (27.8 days), was included in the model to control for variability. Abbreviations: ECG: electrocardiogram; ms: miliseconds.

An ANCOVA analysis with repeated measures was conducted to evaluate the effects of guanfacine dose, the time interval between the first and second ECGs (used as a covariate), and their interactions on the parameters that showed significant differences between the baseline and follow-up evaluations (the QT interval, heart rate, and P wave axis). The analysis revealed a significant effect for the time interval on the changes in the QT interval (*p* = 0.019, partial eta squared = 0.175) (Table 5). The increase in the QT interval was more pronounced in patients receiving higher guanfacine doses (3.00 mg and 4.00 mg) (Figure 2).

## 4. Discussion

This study evaluated the short-term cardiovascular effects of guanfacine treatment over an average exposure period of 27 days in children and adolescents with ADHD. Our findings revealed significant changes in the heart rate, P wave axis, and QT interval during the follow-up period. Specifically, the heart rate decreased by an average of 12.3 bpm (*p* = 0.000), the P wave axis decreased by 12.3° (*p* = 0.038), and the QT interval increased by an average of 16.8 ms (*p* = 0.014). The observed reduction in the heart rate and prolongation of the QT interval are consistent with the cardiovascular effects of guanfacine observed in a previous study [39]. However, the reduction in the P wave axis found in this study has not been previously reported, preventing direct comparisons with prior research. As is consistent with earlier studies, no clinically significant ECG or hemodynamic changes were observed in our study [40].

In our study, we assessed several novel ECG parameters, including the T-wave axis, QRS axis, QRS-T angle, QT dispersion, QTc dispersion, Tp-e interval, Tp-e dispersion, Tp-e/QT ratio, and Tp-e/QTc ratio, that were not previously investigated in the context of guanfacine treatment. These parameters are well established markers of ventricular repolarization and are associated with arrhythmogenesis and cardiac electrical instability in other conditions [35,36]. However, their relevance to guanfacine therapy, particularly in children and adolescents with ADHD, has not yet been explored. In our analysis, no significant changes were observed in any of these parameters between the baseline and follow-up assessments, providing additional evidence of the cardiovascular safety of guanfacine. This novel contribution to the literature highlights the absence of significant effects on advanced markers of electrical activity and supports the continued use of guanfacine as a safe therapeutic option for ADHD.

According to Food and Drug Administration (FDA) reports, guanfacine, which has been prescribed to millions of patients with pre-existing high-risk cardiovascular conditions, is categorized as safe due to its low risk of inducing ventricular arrhythmias and the absence of any guanfacine-associated deaths linked to torsades de pointes [41,42]. A cohort study analyzing 10,927 pediatric cases reported to the U.S. National Poison Data System between 2000 and 2016 for suspected guanfacine toxicity identified the most common side effects as drowsiness, bradycardia, and hypotension [43]; the severity of these side effects was found to increase significantly with higher doses of guanfacine. Moreover, the incidence of major effects was reported as 0.9% across all groups, and a single death was attributed to guanfacine toxicity, although the dose received by the patient was not disclosed. The study also highlighted a broad spectrum of responses to different doses of guanfacine. For instance, in the 0–5 years age group, no clinical effects were observed in patients who received a high dose of 2.72 mg/kg, while major clinical effects were reported in some patients at low doses of 0.05 mg/kg [43]. Considering the average body weight of these patients was approximately 20 kg, this dose corresponds to the initial 1 mg dose commonly prescribed in clinical practice [43]. These findings suggest that the mechanisms underlying guanfacine’s effects and side effects may vary significantly among individuals. Given the wide variability in clinical responses relative to the administered dose, it is recommended that all patients initiated on guanfacine treatment be thoroughly informed about the potential side effects, and informed consent should be obtained when necessary.

In the literature, it was reported that the side effects of guanfacine vary depending on the dosage, with symptoms such as drowsiness/lethargy, bradycardia, hypotension, and QTc prolongation becoming more pronounced at higher doses or in cases of intoxication [43,44]. However, severe cases of hypotension and bradycardia requiring vasopressors or atropine are thought to be rare [43]. In a study by Martin et al., the frequency of sedative events was found to be related to dose, with side effects becoming more frequent as the administered dose increased [45]. Similarly, Knebel et al. investigated the relationship between increasing guanfacine doses and the QTc interval and identified that QTc prolongation was primarily associated with lower body weight and increased exposure to guanfacine due to higher doses of the drug [46]. Therefore, it should be noted that the results observed in our patients when using low-dose guanfacine may differ significantly if the dose is increased to an optimal level. To achieve more objective and comprehensive results, it is necessary to ensure that all patients are treated within the recommended dosing range before re-evaluating the outcomes.

In the study by Hervas et al., reductions in heart rate, systolic BP, and diastolic BP were observed at the 10th week of treatment compared to the baseline values (−3.3 bpm, −2.3 mmHg, and −2.2 mmHg, respectively) [47]. However, no patients discontinued the medication due to these changes, and no clinically significant alterations in QTc interval or ECG findings were reported. Similarly, studies conducted by Biederman [16], Connor [29], and Hirota [19] also demonstrated reductions in heart rate and BP during the acute phase of guanfacine treatment compared to the baseline values. These changes were considered minor and clinically insignificant. As is consistent with previous research, our study also observed reductions in the heart rate (−12.3 bpm), systolic BP (−0.6 mmHg), and diastolic BP (−3.7 mmHg) values compared to the baseline values. However, only the reduction in heart rate was statistically significant. Among our patients, bradycardia and hypotension severe enough to warrant discontinuation of treatment were observed in only two cases. Notably, further advanced cardiological evaluations in these patients revealed no underlying pathology that would contraindicate the continuation of guanfacine. Nonetheless, the families opted to discontinue the medication due to concerns about the symptoms.

Regarding QTc intervals, Hirota et al. [19] observed a mean increase of 5.3 ms from baseline, whereas Sayer et al. [39] reported a decrease of 2 ms. Conversely, Kollins et al. [30] found no significant changes in QTc measurements. In a study by Biederman et al., guanfacine treatment was discontinued in one patient due to a QTc interval exceeding 440 ms [17]. However, the same study observed a mean reduction of 8 ms in QTc values from baseline using Bazett’s formula [16]. A review of the literature revealed no cases in which therapeutic doses of guanfacine led to a QTc increase of 60 ms or more or a QTc interval ≥ 500 ms [16,45,48]. In our study, a significant increase of 16.8 ms in QT interval duration was observed compared to the baseline, whereas QTc showed a non-significant decrease of 0.3 ms. These discrepancies between reports highlight the need for larger, longer-term studies with more extensive sample sizes to further investigate these findings.

### Study Limitations

The small sample size and single-center design may limit the generalizability of the findings. One important limitation of this study is the reduction in the number of participants over the course of the study. Although 65 patients were initially included, 15 were excluded due to meeting the exclusion criteria and 13 were excluded due to a lack of follow-up data. As a result, the final analysis was conducted with 37 patients (56.9% of the initially included cohort), which may have introduced selection bias and affected the study’s representativeness. The focus on short-term outcomes and reliance on a single pediatric cardiology specialist for data evaluation may introduce observer bias. Excluding patients with comorbid psychiatric conditions reduced confounding factors but limited the study’s representation of real-world populations. Additionally, 48.6% of patients underwent cardiac evaluations while on low guanfacine doses, potentially leading to an underestimation of cardiac side effects at higher doses. Another limitation is that some patients received guanfacine in combination with other psychotropic medications. Although these medications were maintained at stable doses, the potential additive or synergistic cardiac effects of agents such as methylphenidate and antipsychotics cannot be entirely ruled out. This factor may have influenced the observed cardiac outcomes and should be considered in future research. Furthermore, in accordance with our hospital’s referral protocol, only patients deemed to be at cardiac risk were referred for pediatric cardiology evaluation before starting guanfacine. As a result, patients without apparent cardiac risk factors were not evaluated by pediatric cardiology, which may have introduced a selection bias and limited the generalizability of our findings to the broader ADHD population. As ADHD diagnoses increase, studies should include patients with pre-existing cardiac conditions to evaluate guanfacine’s safety in this population. Lastly, clinical efficacy was not assessed; future research should explore both efficacy and safety in larger, more diverse cohorts.

## 5. Conclusions

Our study evaluated the short-term cardiovascular effects of guanfacine treatment in children and adolescents with ADHD, and our results align with previous findings. Guanfacine was associated with reduced heart rate and increased QT interval duration, but these changes were not clinically significant. Additionally, we analyzed novel ECG parameters including the T-wave axis, QRS axis, QRS-T angle, QT dispersion, QTc dispersion, Tp-e interval, Tp-e dispersion, Tp-e/QT ratio, and Tp-e/QTc ratio and found no significant differences in response to treatment with guanfacine, supporting its cardiovascular safety. Although guanfacine has been available in Türkiye for two years, more extensive and longer-term studies are needed to address ongoing concerns about its cardiac effects and provide reassurance to clinicians and families, particularly in the context of patients with pre-existing cardiac conditions.

## Figures and Tables

**Table 1:** Descriptive statistics of patients (n = 37).

Parameters		n (%), Median (Min–Max), Mean ± SD
Gender	Girl	9 (24.3%)
	Boy	28 (75.7%)
Age (years)		11.65 (6–16)
Weight (kg)		40 (24–95)
Height (cm)		147 (123–173)
BMI (kg/m^2^)		17.3 (12.76–35.32)
BMI classification	Underweight	6 (16.2%)
	Healthy weight	19 (51.4%)
	Overweight	7 (18.9%)
	Obese	3 (8.1%)
	Severe obesity	2 (5.4%)
Echocardiography results	Normal—insignificant lesions	23 (62.2%)
	Valvular lesions	13 (35.1%)
	Dilatation of the ascending aorta	1 (2.7%)
ADHD		100%
Oppositional defiant disorder		5 (13.5%)
Conduct disorder		2 (5.4%)
Specific learning disorder		12 (32.4%)
Autism spectrum disorder		4 (10.8%)
Intellectual disability		5 (13.5%)
Language and speech disorder		6 (16.2%)
Tic disorder		4 (10.8%)
MPH usage		13 (35.1%)
Atomoxetine usage		1 (2.7%)
Atypical antipsychotic usage		23 (62.2%)
Initial guanfacine treatment	Monotherapy	23 (62.2%)
	Combination therapy (MPH/atomoxetine + guanfacine)	14 (37.8%)
Dose of guanfacine used by patients	1 mg	12 (32.4%)
	2 mg	15 (40.5%)
	3 mg	6 (16.2%)
	4 mg	4 (10.8%)
Guanfacine dosage by weight (mg/kg)		0.05 ± 0.025
Guanfacine dose classification according to weight (n = 37) (the mean weight-adjusted optimal dose)	Optimal dosage	19 (51.4%)
	Low dosage	18 (48.6%)
Time between first ECG and second ECG (n = 37) (days)		27.8 ± 16.8
Side effects		8 (21.6%)

Normally distributed values are expressed as mean ± SD, non-normally distributed values as median (min–max), and groups as n (%).

**Table 2:** Comparison of patient baseline and follow-up data.

Parameters	Groups		*p*
	Baseline Results (n = 37)	Follow-Up Results (n = 37)	
Systolic BP (mmHg)	101.7 ± 14.3	101 ± 13.4	0.838
Diastolic BP (mmHg)	61.8 ± 10.7	57.9 ± 10.3	0.112
Heart rate (bpm)	91 ± 20	79 ± 17	**<0.001**
PR duration (ms)	128 ± 16	127 ± 19.4	0.573
P axis (°)	51 ± 25	40.4 ± 27.9	**0.038**
T axis (°)	47 (1–138)	42 (15–67)	0.089
QRS axis (°)	67 ± 26	67 ± 19	0.697
QRS-t angle (°)	18.7 ± 26.3	25.1 ± 19.5	0.163
QT duration (ms)	352 ± 34.4	370 ± 40	**0.014**
QTc duration (ms)	406 ± 15	406 ± 19.6	0.943
P dispersion (ms)	10 (0–40)	10 (0–40)	0.065
QT dispersion (ms)	20 (0–56)	35 (0–69)	0.395
QTc dispersion (ms)	28 (1–82)	42 (12–367)	0.083
TPe interval (ms)	60 (35–80)	60 (40–80)	0.788
TPe dispersion (ms)	10 (0–40)	10 (0–20)	0.124
TPe/QT ratio	0.17 ± 0.04	0.16 ± 0.03	0.261
TPe/QTc ratio	0.15 ± 0.03	0.14 ± 0.03	0.788

(Mean ± SD for normally distributed data; median (min–max) for non-normally distributed data; groups are written as n (%)). Statistically significant results are in bold.

**Table 3:** Patients with side effects after guanfacine usage.

No	Symptoms Experienced	Frequency of Symptoms
1	Dizziness	2
2	Drowsiness	2
3	Restlessness	2
4	Dizziness, dimming, mild prolongation of the QT interval, and sinus bradycardia	1
5	Hypotension, dizziness	1

**Table 4:** Comparison of patient data before and after guanfacine treatment initiation using paired sample test results.

Pairs (Baseline–Follow-Up Difference)	Paired Differences					*t*	df	Sig. (2-Tailed)
	Mean	Std. Deviation	Std. Error Mean	95% Confidence Interval of the Difference				
				Lower	Upper			
Systolic BP (mmHg)	**0.6**	**16.8**	**2.8**	**−5.0**	**6.2**	0.2	36	0.838
Diastolic BP (mmHg)	3.7	13.7	2.3	−0.9	8.3	1.6	36	0.112
Heart rate (bpm)	12.3	18.5	3.0	6.1	18.5	4.0	36	**<0.001**
P axis (°)	12.3	34.9	5.7	0.7	24.0	2.2	36	**0.038**
QT interval (ms)	−16.8	39.4	6.5	−29.9	−3.6	−2.6	36	**0.014**

Statistically significant results are in bold.

**Table 5:** Analysis of variance (ANCOVA) for changes in electrocardiographic (ECG) parameters considering guanfacine dose and duration of use as covariates.

Measure	Source	Type III Sum of Squares	df	Mean Square	F	*p*	Partial Eta Squared	Box’s Test of Equality of Covariance Matrices
QT interval (ms)	Intercept	2,583,950	1	2,583,950	1888	0	0.99	0.662
	Time interval between the first and second ECG	8398	1	8398	6.1	**0.02**	0.18	
	Guanfacine dose	4955	3	1652	1.2	0.33	0.11	
	Guanfacine dose * time interval between the first and second ECG	3199	3	1066	0.8	0.52	0.08	
Heart rate (bpm)	Intercept	164,414	1	164,414	382	0	0.93	0.298
	Time interval between the first and second ECG	1132	1	1132	2.6	0.12	0.08	
	Guanfacine dose	2382	3	794	1.8	0.16	0.2	
	Guanfacine dose * time interval between the first and second ECG	2598	3	866	2.0	0.13	0.17	
P axis (°)	Intercept	50,275	1	50,275	56.4	0	0.7	0.474
	Time interval between the first and second ECG	26	1	26	0.03	0.87	0.001	
	Guanfacine dose	2489	3	830	0.9	0.44	0.09	
	Guanfacine dose * time interval between the first and second ECG	2518	3	839	0.9	0.43	0.09	

Statistically significant results are highlighted in bold (*p* < 0.005). The asterisk (*) denotes the interaction between Guanfacine Dose and Time Interval Between the First and Second ECG.

## Data Availability

The datasets used and/or analyzed during the current study are available from the corresponding author upon reasonable request due to privacy.

## Abbreviations

The following abbreviations are used in this manuscript:

ADHD	attention deficit/hyperactivity disorder
ANCOVA	analysis of covariance
BMI	body mass index
CGI-S	Clinical Global Impression—Severity
CI	Confidence Interval
DSM-IV	Diagnostic and Statistical Manual of Mental Disorders, Fourth Edition
ECG	electrocardiogram
FDA	Food and Drug Administration
GXR	extended-release guanfacine
K-SADS-PL	Kiddie Schedule for Affective Disorders and Schizophrenia—Present and Lifetime Version
MPH	methylphenidate
QTc	Corrected QT Interval
SCD	sudden cardiac death
SD	standard deviation
WHO	World Health Organization
